# Oxidative Stress and Antioxidant Biomarkers in Clinical and Experimental Models of Non-Alcoholic Fatty Liver Disease

**DOI:** 10.3390/medicina55020026

**Published:** 2019-01-24

**Authors:** Ayokanmi Ore, Oluseyi Adeboye Akinloye

**Affiliations:** 1 Department of Biochemistry, College of Biosciences, Federal University of Agriculture, Abeokuta, Nigeria; oaakin@yahoo.com; 2 Biochemistry Division, Department of Chemical Sciences, Faculty of Natural Sciences, Ajayi Crowther University, Oyo, Nigeria

**Keywords:** liver, NAFLD, NASH, biomarkers, reactive species, oxidative stress, lipid peroxidation, antioxidants

## Abstract

Non-alcoholic fatty liver disease (NAFLD) is a term that covers a range of hepatic disorders involving fat deposits in the liver. NAFLD begins with simple steatosis and progresses into non-alcoholic steatohepatitis (NASH) characterised by inflammation, fibrosis, apoptosis, oxidative stress, lipid peroxidation, mitochondrial dysfunction and release of adipokines and pro-inflammatory cytokines. Oxidative stress and antioxidants are known to play a vital role in the pathogenesis and severity of NAFLD/NASH. A number of oxidative stress and antioxidant markers are employed in the assessment of the pathological state and progression of the disease. In this article, we review several biomarkers of oxidative stress and antioxidants that have been measured at clinical and experimental levels. Also included is a comprehensive description of oxidative stress, sources and contribution to the pathogenesis of NAFLD/NASH.

## 1. Introduction

Non-alcoholic fatty liver disease (NAFLD) is a range of hepatic disorders associated with fatty deposits in liver, which occur in the absence of alcohol consumption or alcohol abuse [1]. NAFLD begins with an initial stage of fatty liver also known as hepatic steatosis (excessive fat loading in the hepatocytes). The progression from steatosis into cirrhosis of the liver due to inflammation and fibrosis results in irreversible damage to the liver [2]. This condition is called non-alcoholic steatohepatitis (NASH)—a term first introduced by Ludwig et al. [3] in clinical subjects with no history of alcohol consumption or abuse.

NAFLD is one of the most common chronic hepatic pathology. It has a worldwide distribution with an estimated prevalence of 25% for NAFLD and about 5% for NASH [4]. The highest prevalence of NAFLD is observed in Western countries (17% to 46%) where it is the most common chronic liver disease (CLD) in adults with a high prevalence of NASH in the United States (16%). The World Gastroenterology Organisation suggests that the prevalence of NAFLD had doubled over the last 20 years. NAFLD and NASH are closely associated with diabetes and obesity, and together are considered one of the major causes of liver disease in Western countries [5].

**Figure 1:**
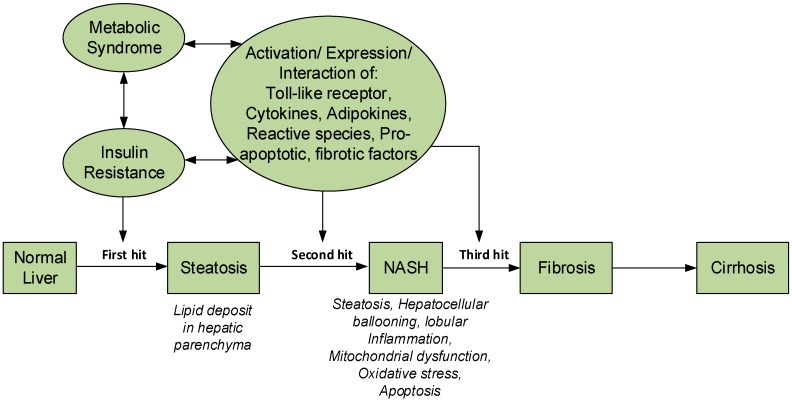
Pathophysiology of NASH and the multi-hit hypothesis.

The pathophysiology of NASH was originally explained by the “two-hit” hypothesis [6]. In this hypothesis, the first hit is responsible for producing steatosis (fat accumulation in liver), and the second hit is from oxidative stress causing lipid peroxidation. The pathogenesis and progression of NAFLD is complex, and was not completely explained by the “two hit” hypothesis. Currently, NASH is described by the “multiple hit” hypothesis [7]. In this hypothesis, metabolic syndrome plays a major role due to insulin resistance and the inflammatory process mediated by interaction of different proteins and immune system. The components of the multiple “hits” are yet to be fully defined and they may vary in different patients. However, from available information, the “first hit” is caused by metabolic syndrome and insulin resistance, increased fat loading in hepatocytes leading to steatosis and liver injury. The accumulation of fat in the liver occur as a result of imbalance between the rate of influx and removal of triglycerides—a mechanism thought to protect hepatocytes from the lipotoxicity that may result from excessive influx of free fatty acids (FFAs) [8]. Most of the FFAs stored as triglycerides originate from increased lipolysis in peripheral tissues as a result of adipose tissue insulin resistance (IR), attended by increased lipogenesis due to hyperinsulinemia and diet fat. The subsequent “hits” are responsible for the inflammation, fibrosis, apoptosis, oxidative stress (OS), and hepatic lipid peroxidation, mitochondrial dysfunction, release of adipokines and pro-inflammatory cytokines [7] (Figure 1). Major histopathological features of NASH are steatosis, hepatocellular ballooning, lobular inflammation and in some cases, fibrosis [7,8] and may progress into cirrhosis if left unchecked.

An understanding of the mechanism of OS, its regulation as well as its role in NAFLD is vital. This will provide researchers in the area of NAFLD/NASH with the best choice of OS/antioxidant (AO) biomarkers useful in pre-clinical investigations as well as in clinical diagnosis of NAFLD. Development of potent drugs in the treatment of NAFLD will also take the AO action required to counteract the OS associated with NAFLD into consideration. In this article, we review the role of oxidative stress in NAFLD and several AO and OS biomarkers that have been measured in pre-clinical and clinical evaluations.

## 2. Oxidative Stress

OS refers to an imbalance between the production of reactive species (RS) and AO defenses [9]. A more encompassing definition describes it as “an imbalance between oxidants and AOs in favour of the oxidants, leading to a disruption of redox signalling and control and/or molecular damage” [9,10]. RS (the oxidants) are chemically reactive species containing oxygen (reactive oxygen species, ROS), or nitrogen (reactive nitrogen species, RNS), etc. (Table 1). ROS (which are the most extensively studied RS) are oxygen-containing molecules that exhibit higher chemical reactivity than oxygen (O_2_). Some ROS are free radicals (e.g., hydroxyl radical, superoxide radical, peroxyl radical etc.), others are non-radicals (e.g., hydrogen peroxide, hypochlorous acid, lipid peroxides etc.). RNS that are free radicals include nitric oxide, nitrogen dioxide etc., and those that are non-radicals include dinitrogen trioxide, peroxinitrite, etc. (Table 1). OS has been classified according to severity as “eustress” (physiological oxidative stress) and “distress” (toxic oxidative burden which damages biomolecules) [10,11]. In other words, low exposure to OS is useful for redox signalling, whereas high exposure results in disruption of redox signalling and causes damage to important biomolecules.

## 3. Oxidative Damage to Macromolecules

OS is associated with many diseases, especially those with an inflammatory mechanism [12]. OS is known in several hepatic diseases with high levels of ROS and RNS, which is an important description of the severity and progression of the disease [13,14]. ROS is constantly generated in the cell due to partial reduction of O_2_ or as a result of transfer of energy to O_2_. ROS can attack vital cell components like polyunsaturated fatty acids, proteins, and nucleic acids, and also carbohydrates in a few cases [15,16,17]. They can disrupt membrane properties like fluidity and ion transport, cause loss of enzyme activity, disruption of the protein synthesis mechanism and induction of DNA damage, ultimately leading to cell death. Damage resulting from OS is often called “oxidative damage”.

Oxidative damage to macromolecules (lipids, proteins, DNA etc.) results in formation of oxidative damage products (Table 2), which are often measured as biomarkers of OS [18,19,20,21]. Some of the most important oxidative damage products of lipids that are frequently measured includes malondialdehyde, lipid peroxides, 8-isoprostane, and 4-hydroxy-2-nonenal (4-HNE). Oxidation of protein can result in protein cross linkage, formation of protein carbonyls, and modification of amino acid. Important products of oxidative modification of amino acids that are frequently measured include 3-nitrotyrosine (a product of ROS-mediated nitration of tyrosine), 2-oxohistidine and hydroxyproline (Table 2). DNA/RNA oxidation can cause single or double strand fragmentation or modification of bases or sugar. 8-Hydroxy-2′-deoxyguanosine (8-OH-dG) and 8-hydroxyguanine (8-OH-G) are the most commonly measured DNA/RNA damage products.

## 4. Regulation of OS

There are several mechanisms for the cellular regulation of OS, which are extremely important to the cell homeostasis. This is achieved through the antioxidant system, which can control the formation of ROS or RNS and also repair oxidative damage to cells. An antioxidant is any substance that can inhibit the oxidation of the cell components such as DNA, proteins and lipids. Several levels of antioxidative defense mechanism are used to prevent oxidative damage [22,23]. The antioxidants can be derived from the diet or endogenously.

Endogenously derived antioxidants are classified as enzymic or non-enzymic antioxidants (Table 3). Enzymic antioxidants of endogenous origin include superoxide dismutase (SOD), catalase (CAT), glutathione peroxidase (GPx), glutathione reductase, etc. SOD catalyse the dismutation of superoxide radical to hydrogen peroxide. The hydrogen peroxide formed in this process and other processes is detoxified by the action of catalase or glutathione peroxidase utilising the power of reduced glutathione (GSH). The latter becomes oxidised (to GSSG) and can be reduced back to GSH through the action of glutathione reductase. Some of the most physiologically vital non-enzymic antioxidants include ascorbate, glutathione, α-tocopherol (vitamin E), Ubiquinone, Thioredoxin (TRX), Bilirubin etc. These antioxidant substances are effective in the detoxification of free radicals and reactive species in the cell (Table 3).

## 5. Sources and Role of OS in NAFLD/NASH

The sources of OS, as well as its role in NASH has been extensively reviewed by Koek et al. [24] and Tariq et al. [25]. OS results from excessive generation of reactive species (RS) or depletion of physiological redox homeostasis. RS (ROS or RNS) from the inflammatory response, the mitochondria, endoplasmic reticulum and peroxisomes also contribute to OS associated with NAFLD/NASH. Their sources and role in NAFLD/NASH are summarized in Table 4.

The mitochondria are important contributors to the OS observed in NAFLD. In the hepatocytes, the mitochondria are not only involved in respiration but they participate in anaplerotic pathways including gluconeogenesis and other biosynthetic activities. During NAFLD, mitochondrial β-oxidation produces ROS that damage hepatocytes contributing to inflammation, and other responses [28]. This increase in ROS production is related to increases in free fatty acid delivery in NAFLD. An increase in fatty acid induces oxidative metabolism, causing an increase in the level of OS and inflammation. The peroxisomal β-oxidation also contributes to the OS in NAFLD through generation of H_2_O_2_. Other sources of OS include the mitochondrial electron transport chain, the activities of microsomal cytochrome P450 enzymes, endoplasmic reticulum stress, the activity of xanthine oxidase and inflammatory responses. Their various contributions to OS in NAFLD are highlighted in Table 4.

OS has been reported to play a significant role in the pathophysiological mechanism of NAFLD and NASH [44,45]. Studies conducted in humans and animal models showed a strong association between the level of OS and the severity of NASH [26,46]. Although clinical and experimental studies have reported higher levels of lipid peroxidation in NASH patients, the levels of circulating antioxidants have been less reported.

## 6. Antioxidant and OS Markers Measured in NAFLD/NASH

Several biomarkers of oxidative stress and antioxidants have been detected in clinical and experimental models of NAFLD and NASH. Most of these are assayed predominantly in the liver, serum, plasma, and in a few cases, in whole blood samples. Major assay procedures for the detection of these markers include colorimetry, ELISA, and immunohistochemistry.

### 6.1. Antioxidant Markers Measured in Clinical NAFLD/NASH

At the clinical level, a number of antioxidants have been determined in the evaluation of NAFLD/NASH. They include the enzymic antioxidants: CAT, SOD, GPx, GR [47,48,49,50,51,52,53,54], and non-enzymic antioxidants: ascorbic Acid, GSH, α-Tocopherol, Ubiquinone, Thioredoxin (TRX) and Bilirubin [48,49,50,51,52,53,54,55,56,57,58] (Table 5). In general, the activities/concentrations of these antioxidants tend to decrease in NAFLD/NASH patients with a few exceptions where they increased. These variations appear to be sample dependent: for instance, those determined in the liver samples of patient generally showed a decrease in their activities/levels in all the data reviewed. However, there are variations with respect to other samples such as blood, plasma and serum, where antioxidant levels tend to increase in most cases of NAFLD and NASH.

### 6.2. Oxidative Stress Markers Measured in Clinical NAFLD/NASH

Oxidative stress biomarkers that have been determined in clinical models of NAFLD include nitric oxide (NO), lipid damage products (lipid peroxides, TBARS (MDA), Hydroperoxides, 8-Isoprostane, 4-HNE), protein oxidation products (protein carbonyl, Nitrotyrosine), DNA oxidation product (8-OH-dG) and CYP2E1 (Table 6). The concentrations/activities of these biomarkers increase generally in all the clinical data reviewed, except where the increase is not significant [52] (Table 6). The most frequently reported OS markers include the NO, MDA, 8-OH-dG and CYP2E1.

### 6.3. Antioxidant Markers Measured in Experimental Models of NAFLD/NASH

Extensive data on antioxidants used to assess NAFLD/NASH in experimental models have been reviewed. They mainly cover the enzymic antioxidants: SOD, CAT, GPx, GR and the non-enzymic antioxidant—GSH in about five rodent models. In nearly all the experimental data reviewed, these antioxidants were measured in the liver and their activities decreased generally in most of the models (mainly NASH), except in a few cases as shown in Table 7.

### 6.4. Oxidative Stress Markers Measured in Experimental Models of NAFLD/NASH

Oxidative stress biomarkers measured at experimental level of NAFLD/NASH include the reactive species (H_2_O_2_, nitrite/nitrate), lipid oxidation products (MDA, Lipid peroxide, 8-Isoprostanes, 4-HNE), protein damage products (Protein carbonyl, Dityrosine, Hydroxyproline, Nitrotyrosine), DNA damage product (8-OH-dG), and oxidative enzymes (CYP2E1, NADPH Oxidase, Xanthine Oxidase), as summarized in Table 8.

The levels or activities of these biomarkers are found to consistently increase in all of the experimental models, except where the increases were not significant. In all the experimental models, MDA is the most measured oxidative stress biomarker as presented in Table 8. Next to MDA is 4-HNE (another product of lipid oxidation), 8-OH-dG and CYP2E1. Others like H_2_O_2_, nitrite/nitrate, lipid peroxide, 8-Isoprostanes, Protein carbonyl, Dityrosine, Hydroxyproline, Nitrotyrosine), NADPH Oxidase and Xanthine Oxidase are not frequently measured. However, their levels/activities increase in a similar pattern to MDA, 8-OH-dG, 4-HNE, CYP2E1.

## 7. Conclusions

Oxidative stress plays an important role in the pathophysiology of NAFLD/NASH. Several markers of oxidative stress and antioxidants have been shown to be very useful in assessing the redox state in NAFLD/NASH. Among the oxidative stress biomarkers reviewed, TBARS, MDA, CYP2E1 and 4-HNE are unique; they are represented in both clinical and experimental measurements and they consistently increase. Antioxidants of interest in clinical and pre-clinical assessment of NAFLD/NASH include GSH, SOD, CAT, and GPx, which appear to be most reliably detected in liver samples.

## Figures and Tables

**Table 1:** Major ROS and RNS of physiological importance.

**Major ROS**		**Sources**
Free Radicals	Hydroxyl radical ($OH^{•}$)	Decomposition of ONOO^−^ or, HOCl
	Superoxide radical ($O_{2}^{•-}$)	Electron transport systems, and one-electron reduction of O_2_ by respiratory burst via the action of membrane bound NADPH oxidase
	Peroxyl radical (ROO^•^)	Produced in the Fenton reaction
Non-Radicals	Hydrogen peroxide (H_2_O_2_)	Activated macrophages during inflammation
	Hypochlorous acid (HClO)	Combined activities of NADPH oxidase and myeloperoxidase (MPO) in phagocytes
	Lipid peroxides (ROOH)	Formed from oxidation of polyunsaturated fatty acid via lipid-peroxyl radical reaction
**Major RNS**		**Sources**
Free Radicals	Nitric oxide (NO^•^)	Nitric Oxide Synthase (NOS)
	Nitrogen dioxide ($NO_{2}^{•}$)	Activated neutrophils
Non-radicals	Dinitrogen trioxide (N_2_O_3_)	Produced in pathological conditions where (inducible nitric oxide (iNOS) is upregulated
	Peroxinitrite (ONOO^−^)	Produced in pathological conditions where iNOS is upregulated
	Nitrite ($NO_{2}^{-}$)	Oxidation product from NO, formed during NOS activation in inflammatory diseases
	Nitryl ion ($NO_{2}^{+}$)	Activated neutrophils

**Table 2:** Some important oxidative damage products.

Macromolecule	Oxidative Damage	Damage Products
Lipids	Lipid oxidation/peroxidation	Malondialdehyde (MDA) Lipid peroxide, (lipid endoperoxides and lipid hydroperoxides) 8-Isoprostane 4-hydroxy-2-nonenal (4-HNE)
Proteins	Protein Oxidation, protein cross linkage, Oxidative modification of amino acids	Protein carbonyl compounds, 3-Nitrotyrosine (product of ROS-mediated nitration of tyrosine), 2-oxohistidine, hydroxyproline etc.
DNA	RNA/DNA fragmentation (single and double-strand breaks) Modification of base, sugar	8-hydroxy-2′-deoxyguanosine (8-OH-dG), 8-hydroxyguanine (8-OH-G)

**Table 3:** Physiologically important antioxidants and their functions.

Antioxidant Type	Antioxidant Name	Functions
Enzymic antioxidants	Superoxide dismutase (SOD)	Converts $O_{2}^{•-}$ to H_2_O_2_ and O_2_
	Catalase (CAT)	Converts H_2_O_2_ to 2H_2_O and O_2_
	Glutathione peroxidase (GPx)	Detoxifies H_2_O_2_ and Lipid peroxides using reduced glutathione (GSH) producing the oxidised form of glutathione (GSSG)
	Glutathione reductase (GR)	Reduces GSSG to GSH
Non-enzymic antioxidants	Ascorbic Acid (AA)	Detoxifies Superoxide radical, Hydroxyl radical, and H_2_O_2_
	Reduced Glutathione (GSH)	Neutralizes Superoxide radical, Hydroxyl radical, and H_2_O_2_; co-substrate for glutathione peroxidase
	α-Tocopherol	Detoxifies H_2_O_2_; protects against membrane lipid peroxidation (LPO)
	Ubiquinone	Detoxifies Lipid peroxides
	Thioredoxin (TRX)	General thiol redox control of protein activity via reversible disulfide formation
	Bilirubin	Effective in quenching/scavenging secondary oxidants produced during OS

**Table 4:** Sources of oxidative stress in NAFLD/NASH.

Source	Contribution to OS in NAFLD/NASH	References
Mitochondrial metabolism (β-oxidation)	Production of ROS as a result of electron leakage during mitochondrial β-oxidation	[26,27,28,29,30]
Peroxisomal β-oxidation	Generation of H_2_O_2_ during peroxisomal β-oxidation which is converted into hydroxyl radical contributing to OS	[31]
Mitochondrial electron transport chain	inhibition of electron transport chain by TNF-α and lipid peroxidation products	[32,33]
Microsomal Cytochrome P450 enzymes	ROS generation due to Increase in activity of cytochrome P4502E1 (CYP2E1) involved in lipooxygenation of longchain fatty acids	[34,35,36,37]
Endoplasmic reticulum (ER) stress	Endoplasmic reticulum stress response, promotes OS via increased expression of CHOP (also called DDIT –DNA Damage Inducible Transcript-3 protein)	[38]
Xanthine Oxidase (XO)	Increase in XO activity generates superoxide anions, due to induction by 4-HNE (a product of lipid peroxidation)	[39,40]
Inflammatory Response	Abnormal inflammatory response mediated by gut microflora resulting in increase in pro-oxidants	[41,42,43]

**Table 5:** Antioxidant markers measured in clinical NAFLD/NASH.

Antioxidant Marker	Sample	Level/Activity/Expression in Sample	Clinical Case	Reference(s)
**SOD**	Serum	Decreased, Increased (nsc)	NASH	[47,48,49]
	Serum/Liver	Decreased	NAFLD	[50]
	Plasma	Decreased	NAFLD	[51]
	Blood	Increased	NAFLD	[52]
	Liver	Decreased	NAFLD	[53]
	Blood	Increased	NAFLD	[54]
**CAT**	Serum	Decreased, Increased (nsc)	NASH	[47,48]
	Plasma	Decreased	NAFLD	[51]
	Blood	Increased	NAFLD	[52]
	Liver	Decreased	NAFLD	[53]
	Blood	Decreased	NAFLD	[54]
**GPx**	Serum	Decreased	NASH	[48,49]
	Blood	Increased	NAFLD	[54]
	Liver	Decreased	NAFLD	[53]
**GR**	Serum	Increased	NASH	[49]
	Blood	Increased	NAFLD	[54]
**Ascorbic Acid**	Serum	nsc	NASH	[48]
	Serum	Decreased	NAFLD	[50]
**GSH**	Serum	Increased	NASH	[49]
	Blood	Increased	NAFLD	[52]
	Liver	Decreased	NAFLD	[50,53]
	Blood	Decreased	NAFLD	[54]
**α-Tocopherol**	Serum	Increased	NASH	[48]
	Serum	Decreased	NAFLD	[50]
**Ubiquinone**	Serum	Decreased	NAFLD	[51]
**Thioredoxin (TRX)**	Serum	Increased	NAFLD	[55]
**Bilirubin**	Serum	Decreased	NASH	[56,57]
	Serum	Decreased	NAFLD	[58]

nsc: no significant change.

**Table 6:** Oxidative stress markers measured in clinical NAFLD/NASH.

OS Marker	Sample	Level/Activity/Expression in Sample	Clinical Case	Reference(s)
Lipid peroxides	Plasma	Increased	NASH	[59]
NO^•^	Serum	Increased	NASH	[47,49]
	Serum	Increased	NAFLD	[50,60]
	Blood	Increased	NAFLD	[52]
TBARS/MDA	Serum	Increased	NAFLD	[47]
	Serum	Increased	NAFLD/NASH	[49,51,61]
	Serum	Increased	NAFLD	[50]
	Blood	Increased	NAFLD	[54]
Hydroperoxides	liver	Increased	NASH	[62]
8-Isoprostane	Plasma	Increased	NASH	[48]
4-HNE	Liver	Increased	NASH	[63]
Protein carbonyl	Liver	Increased	NAFLD	[53]
Nitrotyrosine	Blood	nsc	NAFLD	[52]
8-OH-dG	Liver	Increased	NASH	[63,64]
	Liver	Increased	NAFLD	[65]
CYP2E1	Liver	Increased	NASH	[34,35]
	Liver	nsc	Steatosis/NASH	[66]
	Liver	Increased	NAFLD	[53]

nsc: no significant change.

**Table 7:** Antioxidant markers measured in experimental NAFLD/NASH.

Antioxidant Marker	Sample	Level/Activity/Expression in Sample	Experimental Model	Experimental Specie	Reference(s)
SOD	Liver	Decreased	NASH (MCD)	Wistar Rats	[67]
	Liver	Increased	NASH (MCD)	C57BL/6 Mice	[68]
	Liver	Increased	NASH (MCD)	C57BL/6 mice	[69]
	Liver	Decreased	NASH (MCD)	C57BL6/J mice	[70]
	Liver	Decreased	NASH (MCD)	N-Mary rats	[71]
	Liver	Decreased	NAFLD (HFD)	Mice	[72]
	Liver	Decreased	NASH (HF)	Kunming mice	[73]
	Liver	Increased	NAFLD (HFD)	Rat	[74]
CAT	Liver	Decreased	NASH (MCD)	Wistar Rats	[67]
	Liver	Decreased	NASH (MCD)	C57BL/6 Mice	[68]
	Liver	Increased	NASH (MCD)	C57BL/6 mice	[69]
	Liver	Decreased	NASH (HCD)	Wistar Rats	[75]
	Liver	Decreased	NAFLD (HFD)	Sprague-Dawley rats	[74]
GPx	Liver	Decreased	NASH (MCD)	Wistar Rats	[67]
	Liver	Increase	NASH (MCD)	N-Mary rats	[71]
	Liver	Decreased	NAFLD (HFD)	Mice	[72]
	Liver	Decreased	NASH (HF)	Kunming mice	[73]
	Liver	nsc	NAFLD (HFD)	Sprague-Dawley rats	[74]
GR	Liver	Decreased	NASH (MCD)	N-Mary rats	[71]
GSH	Liver	Decreased	NASH (MCD)	Wistar Rat	[67]
	Liver	Decreased	NASH (MCD)	N-Mary rats	[71]
	Liver	Decreased	NAFLD (HFD)	Wistar Rats	[76]
	Liver	Decreased	NAFLD (HCD)	Wistar Rats	[75]
	Liver/RBC	Increased	NASH (HF-MCD)	Sprague-Dawley rats	[77]
	Liver	Decreased	NASH (MCD)	Mice	[78]

MCD: Methionine/Choline Deficient Diet; HF: High fructose Diet; HFD: High Fat Diet; HCD: High Cholesterol diet; HF-MCD: High fat- methionine choline deficient diet; nsc: no significant change.

**Table 8:** Oxidative stress markers measured in experimental NAFLD/NASH.

OS Marker	Sample	Level/Activity/Expression in Sample	Experimental Model	Experimental Specie	Reference(s)
H_2_O_2_	Liver	Increased	NASH (MCD)	C57BL/6J-mt^FVB/N^ mice	[79]
Nitrite/nitrate	Liver	Nsc	NAFLD (HFD)	Wistar Rats	[76]
TBARS (MDA)	Liver	Increased	NASH	Rat	[80]
	Liver	Increased	NASH (MCD)	C57BL/6 Mice	[70]
	Liver	Increased	Steatosis/NASH (HFD)	Albino rats	[81]
	Liver	Increased	NASH (MCD)	C57BL/6 mice	[69]
	Liver	Decreased	NAFLD (HCD)	Wistar Rats	[75]
	Liver	Increased	Steatosis (HFD/HSD)	Wistar rats	[82]
	Liver	Increased	HF/HGD	Wistar rats	[83]
	Liver	Increased	NAFLD (HFD)	C57BL/6J mice	[84]
	Liver	Increased	NASH (MCD)	C57BL/6 mice	[85]
	Liver	Increased	NAFLD/NASH (CDAA diet)	Wistar Rats	[86]
	Liver	Increased	NAFLD (HFD)	Sprague-Dawley rats	[74]
	Liver	Increased	NASH (MCD)	C57BL6/J mice	[70]
	Liver	Increased	NASH	Rat	[80]
	Liver	Increased	NAFLD (HFD)	Wistar Rats	[76]
	Liver	Increased	NASH (CDHF diet)	Wistar Rats	[87]
	Liver	Increased	NASH (MCD)	N-Mary rats	[71]
	Liver	Increased	NAFLD (HFD)	Mice	[72]
	Liver	Increased	NASH (HF)	Kunming mice	[73]
	Liver	Nsc	NASH (MCD)	C57BL/6J-mt^FVB/N^ mice	[79]
Lipid peroxide	Liver	Increased	NASH (MCD)	Wistar Rat	[67]
8-Isoprostanes	Liver	Increased	NASH (HFMCD)	Sprague Dawley Rat	[77]
4-HNE	Liver	Increased	NASH (MCD)	Wistar Rat	[67]
	Liver	Increased	NASH	Rat	[80]
	Liver	Increased	NASH (HFD)	Sprague Dawley Rat	[88]
	Liver	Increased	NASH (CDHF diet)	Wistar Rats	[87]
	Liver	Increased	NASH	leptin-deficient (ob/ob) mice	[89]
	Liver	Increased	NASH (HF-HSD)	C57BL/6 J mice	[90]
	Liver	Increased	NASH (MCD)	C57BL6 mice	[54,91,92]
Protein carbonyl	Liver	Increased	NASH (MCD)	N-Mary rats	[93]
Nitrotyrosine	Liver	Increased	NAFLD	CYP2E1 transgenic (Tg) mice	[93]
Dityrosine	Liver	Increased	NAFLD (HFD)	C57BL/6J mice	[84]
Hydroxyproline	Liver	Increased	NAFLD/NASH (CDAA diet)	Wistar Rats	[86]
	Liver	Increased	NASH (MCD/WD)	C57BL6 mice	[54]
8-OH-dG	Liver	Increased	NASH (MCD)	Wistar Rat	[69]
	Liver	Decreased	NASH (HF-MCD)	Sprague-Dawley rats	[79]
	Liver	Increased	NAFLD (HFD)	C57BL/6J mice	[86]
CYP2E1	Liver	Increased	NAFLD	CYP2E1 transgenic (Tg) mice	[92,93]
	Liver	Increased	NASH (HFD)	Sprague Dawley Rat	[88]
	Liver	Increased	NASH (HFD)	Sprague Dawley Rat	[94]
	Liver	Increased	Steatosis (HFD/HSD)	Wistar rats	[82]
	Liver	Increased	NASH (HFD)	Sprague-Dawley rats	[95]
	Liver	Increased	NASH (CDHF diet)	Wistar rats	[96]
NADPH Oxidase	liver	Increased	NASH	ob/ob mice	[89]
Xanthine Oxidase	Liver	Increased	NAFLD (HFD)	Sprague-Dawley rats	[74]

MCD: Methionine/Choline Deficient Diet; HF: High fructose Diet; HFD: High Fat Diet; HCD: High Cholesterol diet; HSD: High Sucrose diet; CDAA: Choline Deficient L-Amino Acid-defined; CDHF: Choline Deficient High Fat diet; HFMCD: High fat methionine choline deficient diet; HF-HSD: High fat-high sucrose diet; WD: Western diet; HF/HGD: High-Fructose/High-Glucose Diet; nsc: no significant change.
